# Olfactory marker protein is unlikely to be cleaved by calpain 5

**DOI:** 10.1186/s13041-022-00971-2

**Published:** 2022-10-29

**Authors:** Noriyuki Nakashima, Kie Nakashima, Akiko Nakashima, Makoto Takano

**Affiliations:** 1 Department of Physiology, Kurume University School of Medicine, 67 Asahi-machi, 830-0011 Kurume- shi, Fukuoka, Japan; 2 Department of Physiology and Cell Biology, Kobe University School of Medicine, Chuo, Kobe Japan

**Keywords:** Olfactory marker protein (OMP), Calpain 5 (CAPN5), Reciprocal expression, Ca^2+^-dependent protease

## Abstract

Olfactory maturation marker protein (OMP) is expressed in olfactory receptor neurons and hypothalamic neurons. OMP is a nested gene located in the intron of calpain 5 (CAPN5), a Ca^2+^-dependent cysteine protease. Despite being located at the same genomic locus, genetic regulation of the reciprocal expression of OMP and CAPN5 has been suggested. By performing a motif search, we detected possible calpain cleavage sites in OMP. However, the direct proteolytic regulation of OMP by CAPN5 is unclear. Here, we generated OMP fused with Myc-tag and His-tag at its N- and C-termini and examined whether CAPN5 cleaves OMP into fragments by detecting immunoreactivity against Myc, OMP and His. Western blotting demonstrated that OMP was unlikely to be cleaved even in the presence of Ca^2+^ in vitro. We expressed OMP and CAPN5 in HEK293T cells and applied a calcium ionophore under physiological conditions *in cellulo*, which resulted in no apparent fragmentation of OMP. We also applied liquid chromatography/mass spectrometry to the electrophoresed fractions smaller than the uncut Myc-OMP-His signals, which demonstrated no significant fragmentation of OMP. These results collectively indicate that OMP is unlikely to be cleaved by CAPN5.

**Supplementary Information:**

The online version contains supplementary material available at 10.1186/s13041-022-00971-2.

## Introduction

OMP is a maturation marker of olfactory receptor neurons important for olfaction [1]. OMP is also expressed in the hypothalamus [2, 3], where OMP expression is restricted in a limited subset of cells. OMP is a nested gene located in the intron of calpain 5 (CAPN5). Expressed from the same locus, OMP and CAPN5 were reciprocally detected in the olfactory bulb and the hypothalamus [3] (Supplementary Fig. 1a-c). The promoter is sufficient to induce olfactory-specific expression of OMP [4]. However, the negative regulation of OMP expression is not well explained [5]. Among the calpain family members, CAPN5 requires extremely high Ca^2+^ concentrations to exert maximal activity [6, 7]. Therefore, we hypothesized that OMP could be cleaved into fragments [8] by Ca^2+^-activated CAPN5 under a large Ca^2+^ load. Here, we detected several possible calpain cleavage sites in OMP and examined whether OMP is fragmented by CAPN5 in the presence of Ca^2+^ in vitro and *in cellulo*.

## Results

**Fig. 1:**
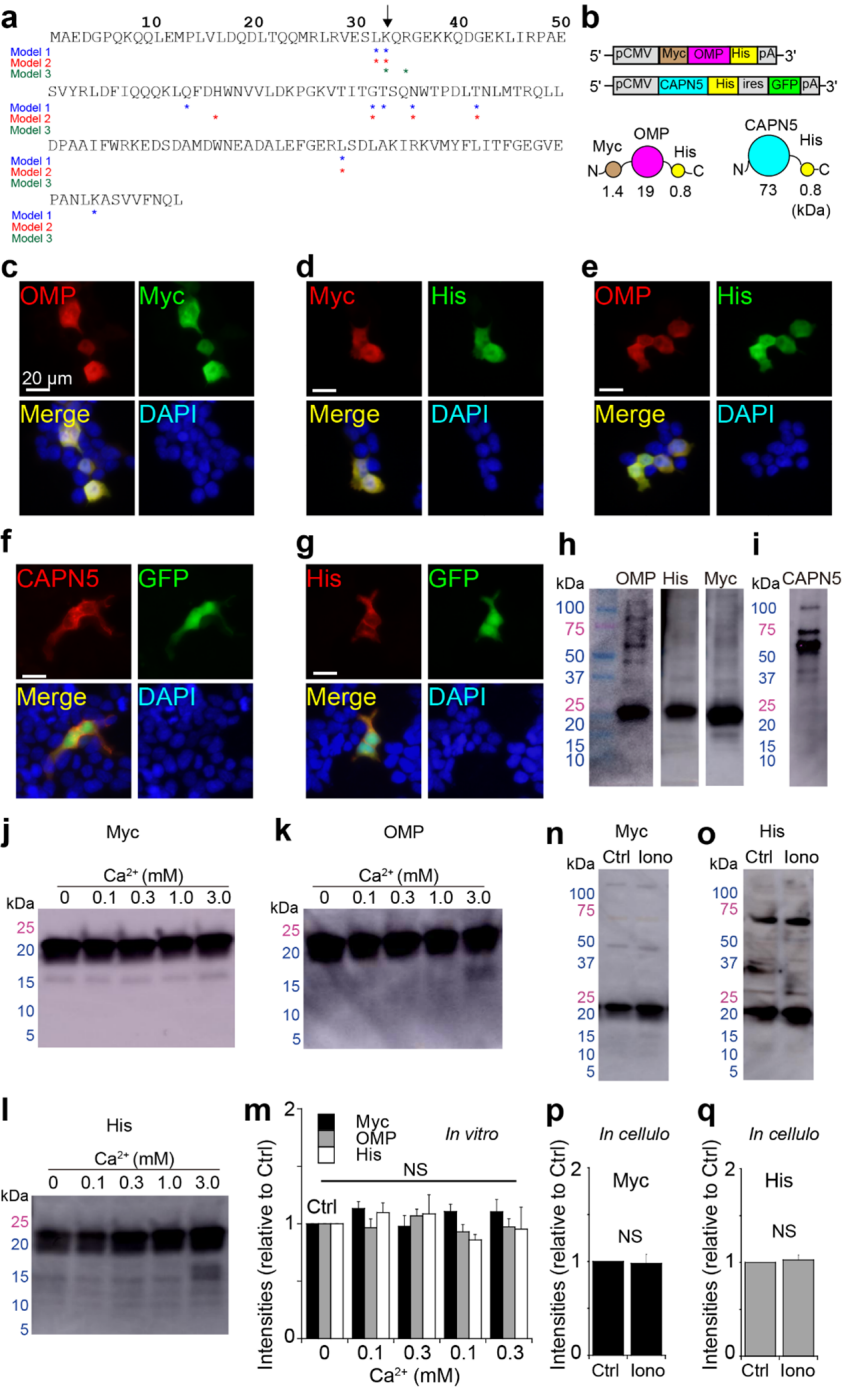
**OMP was unlikely to be cleaved by CAPN5.** **a**, Primary structure of mouse OMP. The asterisks indicate the highly scored residues in three prediction models: Model 1, support vector machine (SVM) with radial basis function kernel; Model 2, SVM with linear kernel; Model 3, protein secondary structure prediction. See Methods for details. All models predict a possible cleavage at K33 (arrow). **b**, Plasmids and molecular diagrams for Myc-OMP-His and CAPN5-His with approximate molecular weights in kDa. N and C; N- and C-termini; ires, internal ribosomal entry site. **c-e**, Confirmation of concomitant immunoreactivity (IR) to the fusion myc-OMP-His protein expressed in HEK293T cells: (**c**) OMP-IR and Myc-IR; (**d**) Myc and His-IR; (**e**) OMP-IR and His-IR. **f,g**. CAPN5 and His expression was confirmed by GFP coexpression because the antibodies were both raised in mice: (**f**) CAPN5-IR and GFP fluorescence and (**g**) His-IR and GFP fluorescence. Scales, 20 μm in (**c-g**). DAPI, 4’,6-diamidino-2-phenylindole. **h, i**, Confirmation of purification of His-tagged purified (h) Myc-OMP-His (22 kDa) and (**i**) CAPN5-His (74 kDa) by western blotting. **j-l**, Size of Myc-OMP-His by (**j**) Myc-IR, (**k**) OMP-IR and (**l**) His-IR after incubation with CAPN5-His in the presence of different concentrations of Ca^2+^. No apparent fragmentation of OMP was observed below 22 kDa. Note that cleavage at K33 in OMP should result in two fragments: 5 kDa (Myc-tagged N-terminus) and 16 kDa (His-tagged C-terminus). **m**. Summary of the western blot intensities for Myc-, OMP- and His-IRs (n = 4). No significant changes in these intensities were detected. **n, o**, Western blot of the cell lysate of HEK293T cells expressing (**n**) Myc-OMP-His and (**o**) CAPN5-His in the absence (Ctrl) and presence of Ca^2+^-ionophore (1 µM; Iono) for 12 h. **p, q**, Summary of western blot intensities for (**p**) Myc-IR and (**q**) His-IR (n = 3). No smaller fragments were observed below 22 kDa, corresponding to the uncut Myc-OMP-His. Mean ± SD. **STATISTICS**: (**m**) One-way ANOVA with a *post hoc* Tukey‒Kramer comparison among different Ca^2+^-concentration groups (0–3 mM): Myc; F(4,19) = 1.185, P = 0.35696: His; F(4,19) = 0.884, P = 0.497: OMP; F(4,19) = 0.977935, P = 0.44871. No significant difference was detected (NS). The actual values from *the post hoc* test are shown in the Supplementary Data. (**p,q**) Paired, two-tailed Student’s T test for the comparison between Ctrl and Iono; P = 0.821 for Myc, P = 0.6 for His.

The consensus sequence for calpain cleavage remains unclear [9]. Thus, we consulted public prediction software (Methods). Several possible residues were cleaved by calpains [9–11]. Three different models listed K33 as a possible candidate (Fig. 1a).

To detect the N- and C-terminal fragments in the subsequent experiments, we tagged OMP with Myc-tag (Myc) and 6xHistidine-tag (His) (22.1 kDa, Fig. 1b). To purify the proteins by His-column, CAPN5 was also fused with His (Figs. 1b and 73.8 kDa). We transfected cDNAs into HEK293T cells and confirmed that OMP-IR was simultaneously detected with Myc- and His-IRs (Fig. 1c-e). Because the available antibodies for CAPN5 and His-tag were both raised in mice, we subcloned CAPN5-His into a GFP-coexpressing vector and confirmed that CAPN5-IR and His-IR both colocalized with GFP fluorescence (Fig. 1f, g; Supplementary Fig. 2a-g), indicating that CAPN5 and His-tag were successfully fused.

To directly assess whether OMP was prone to CAPN5 cleavage, we purified the tagged proteins by using a His-tag affinity column. The collected proteins were resuspended in divalent cation-free phosphate-buffered saline (PBS(-)). The sizes and concentrations of purified proteins were confirmed by Myc-, OMP-, His-, CAPN5-IRs prior to mixing two proteins (Fig. 1 h, i). Myc-OMP-His (22.1 kDa) at 1 µg/µL and CAPN5-His (73.8 kDa) at 0.1 µg/µL were mixed in PBS(-) and incubated in the presence of Ca^2+^ (0–3 mM) for 24 h. By western blotting, Myc-, OMP- and His-IRs were all detected in the 22 kDa band corresponding to noncleaved Myc-OMP-His (Fig. 1j-l), and the signal intensities were unchanged in the absence or presence of Ca^2+^ (Fig. 1 m). The enzymatic activities of CAPN5 were assessed by the autolytic efficacy of CAPN5 as previously adopted [12, 13] due to the lack of information on CAPN5 substrates [13]. CAPN5-His-IR was diminished in the presence of Ca^2+^, indicating that CAPN5-His was subject to autocleavage by its own enzymatic activity (Supplementary Fig. 3a,b) [12, 13].

To eliminate the possibility that PBS(-) affected the enzymatic activity of CAPN5, we expressed Myc-OMP-His and CAPN5-His in HEK293T cells and applied the Ca^2+^-ionophore ionomycin. However, Myc-IR or His-IR for OMP were similarly detected in the 22 kDa band both in the absence (control) and presence of ionomycin, indicating that OMP was uncleaved by CAPN5 (Fig. 1n-q). The tag peptides could interfere with the cleavage of OMP, and we expressed the native form of OMP together with CAPN5-His in HEK293T cells. Ionomycin induced no significant decrease in OMP-IR (Supplementary Fig. 4a,b).

Last, we applied liquid chromatography/mass spectrometry (LC/MS) to the electrophoresed bands below approximately 17 kDa in Myc-OMP-His alone or with CAPN5-His in the absence or presence of 3 mM Ca^2+^ (Supplementary Fig. 5). No significant fragmentation of OMP was detected.

## Discussion

Our results indicate that OMP is not apparently cleaved by CAPN5 even in the presence of Ca^2+^; 3 mM Ca^2+^, which was far above the physiological intracellular concentration, did not facilitate OMP cleavage. CAPN5 was reported to require Ca^2+^ in the millimolar range to exert its maximal activity [6, 7, 13]. Extremely high concentrations of Ca^2+^ may transiently occur near the endoplasmic reticulum or membrane microdomains, but the cleavage efficacy of OMP might be negligible in such short time periods. Apart from the efficacy of cleavage, the accessibility of CAPN5 to OMP remains unsolved. Although the cleavage candidate residues and Myc/His-tags were mutually situated apart within Myc-OMP-His (Supplementary Fig. 6a,b), the steric hindrance by Myc/His-tags cannot be fully denied. Meanwhile, the native form of OMP was unaffected by CAPN5 with Ca^2+^ load in HEK293T cells. Therefore, we conclude that the reciprocal expression of OMP and CAPN5 [3] is unlikely to be due to the CAPN5-dependent cleavage of OMP. OMP and CAPN5 might be expressed from the same locus under any circumstance, and the extremely high requirement for Ca^2+^ of CAPN5 should act as a safeguard to prevent the breakdown of OMP [7].

OMP is proposed to enter the nucleus in association with the transcription factor Bex1 and may play a role in gene expression [2, 14] and to participate in regulation of the differentiation of olfactory receptor neurons [14]. In the brain, OMP may also affect the transcriptional properties of hypothalamic neurons [15]. Considering that only a limited subset of hypothalamic neurons express OMP [2], the turnover mechanisms of OMP need to be investigated in parallel with the expression mechanisms [4], subcellular localization [2] and cellular conditions [13].

## Electronic supplementary material

Below is the link to the electronic supplementary material.

Supplementary Material 1

Supplementary Material 2

## Data Availability

All data generated or analysed during this study are included in this published article and its additional files.

## Abbreviations

CAPN5: calpain 5
His: 6xHistidine-tag
IR: immunoreactivity
LC/MS: liquid chromatography/mass spectrometry
Myc: Myc-tag
OMP: olfactory maturation marker protein
PBS(-): phosphate-buffered saline
SVM: support vector machine
