# Supplementary material for: Olfactory marker protein is unlikely to be cleaved by calpain 5
Source: Mol Brain. 2022 Oct 29;15:87. doi: 10.1186/s13041-022-00971-2 (PMC9618205; doi:10.1186/s13041-022-00971-2)
Supplement: Supplementary file 2 — Supplementary Material 2 [file 13041_2022_971_MOESM2_ESM.docx]

**Supplementary information for**

**Title: Olfactory marker protein is unlikely to be cleaved by calpain 5**

**Authors:** Noriyuki Nakashima^1*#^, Kie Nakashima^2*#^, Akiko Nakashima^1*#^, Makoto Takano^1^

**Affiliations:**

^1^ Department of Physiology, Kurume University School of Medicine, 67 Asahi-machi, Kurume-shi, Fukuoka, 830-0011, Japan.

^2^ Department of Physiology and Cell Biology, Kobe University School of Medicine, Chuo, Kobe, Japan.

**Contact information:**

Noriyuki Nakashima: [nakashima_noriyuki@med.kurume-u.ac.jp](mailto:nakashima_noriyuki@med.kurume-u.ac.jp)

Kie Nakashima: [nakasima@med.kobe-u.ac.jp](mailto:nakasima@med.kobe-u.ac.jp)

Akiko Nakashima: [nakashima_akiko@med.kurume-u.ac.jp](mailto:nakashima_akiko@med.kurume-u.ac.jp)

Makoto Takano: [takanom@med.kurume-u.ac.jp](mailto:takanom@med.kurume-u.ac.jp)

* **Correspondences**:

Noriyuki Nakashima: [nakashima_noriyuki@med.kurume-u.ac.jp](mailto:nakashima_noriyuki@med.kurume-u.ac.jp)

Kie Nakashima: [nakasima@med.kobe-u.ac.jp](mailto:nakasima@med.kobe-u.ac.jp)

Akiko Nakashima: [nakashima_akiko@med.kurume-u.ac.jp](mailto:nakashima_akiko@med.kurume-u.ac.jp)

Phone: +81-942-31-7543

FAX: +81-942-31-7728

**# Equal contributions**

**Supplementary Figure 1**


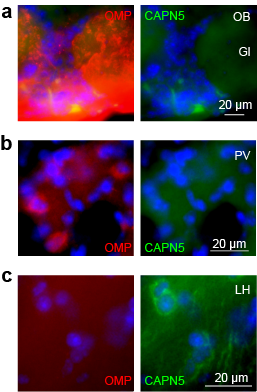


**Legend for Supplementary Figure 1**

**OMP and CAPN5 were exclusively expressed in olfactory receptor neurons and hypothalamic neurons.**

**a,** Terminals of ORNs with OMP-IR expression in the olfactory glomeruli (Gl) showed no apparent CAPN5-IR expression in the olfactory bulb (OB). Some periglomerular cells showed expression of CAPN5-IR, which was embedded in the axon layer of the OB. **b,** Hypothalamic neurons with OMP-IR expression showed no apparent CAPN5-IR expression in the periventricular nuclei (PV). **c,** Hypothalamic cells with CAPN5-IR expression showed no OMP-IR expression in the lateral hypothalamus (LH). Blue, nucleus stained with 4',6-diamidino-2-phenylindole.

**Supplementary Figure 2**

**
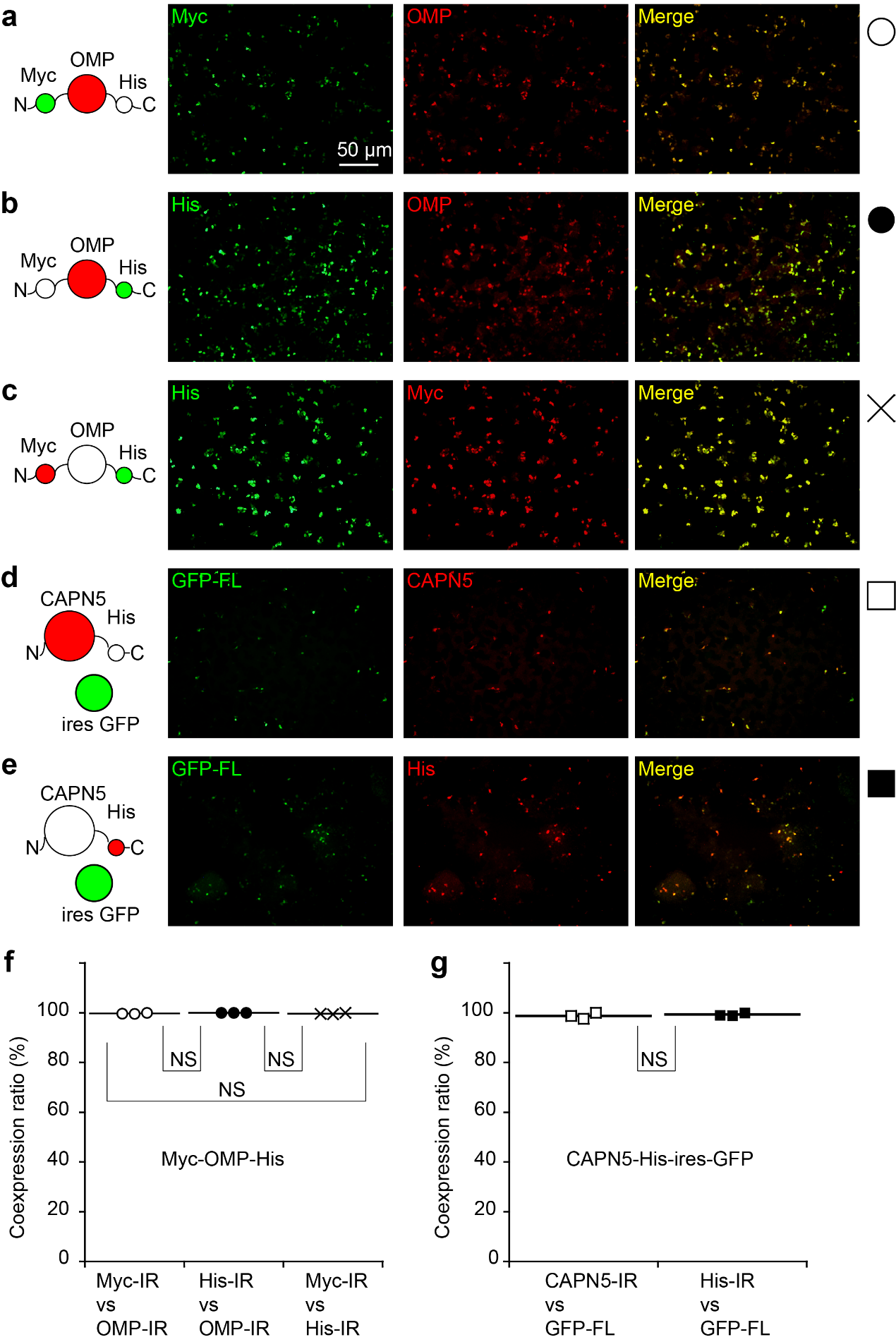
**

**Legend for Supplementary Figure 2**

**The generation of Myc-OMP-His and CAPN5-His was confirmed in HEK293T cells.**

**a-c,** Immunoreactivities in the fusion protein Myc-OMP-His against (a) Myc and OMP, (b) His and OMP and (c) Myc and His. **d, e,** Immunoreactivities in CAPN5-His against (d) CAPN5 and (e) His with coexpression of GFP-fluorescence (GFP-FL) under the control of ires. The models of the fusion proteins and the observed fluorescent colours are shown on the left. The symbols on the right correspond to the graph data in (f, g). **f, g,** No significant difference between immunoreactivities and reporter fluorescence was detected in the fusion proteins (f) Myc-OMP-His and (g) CAPN5-His-ires-GFP.

**STATISTICS: (f, g)** One-way ANOVA with a *post hoc* Tukey‒Kramer comparison among different immunoreactivities and fluorescence; (f) Myc-OMP-His, F(2,6)=2.00, P=0.216, Tukey-Kramer test, P_(Myc/OMP vs His/OMP)_=0.413, P_(Myc/OMP vs His/Myc)_=0.836, P_(His/OMP vs His/Myc)_=0.206; (g) CAPN5-His-ires-GFP, F(2,6)=0.6101, P=0.478, Tukey‒Kramer test, P_(His/GFP vs CAPN5/GFP)_ =0.478. No significant difference was detected (NS), indicating that the tags were successfully fused to OMP and CAPN5.

**Supplementary Figure 3**

**
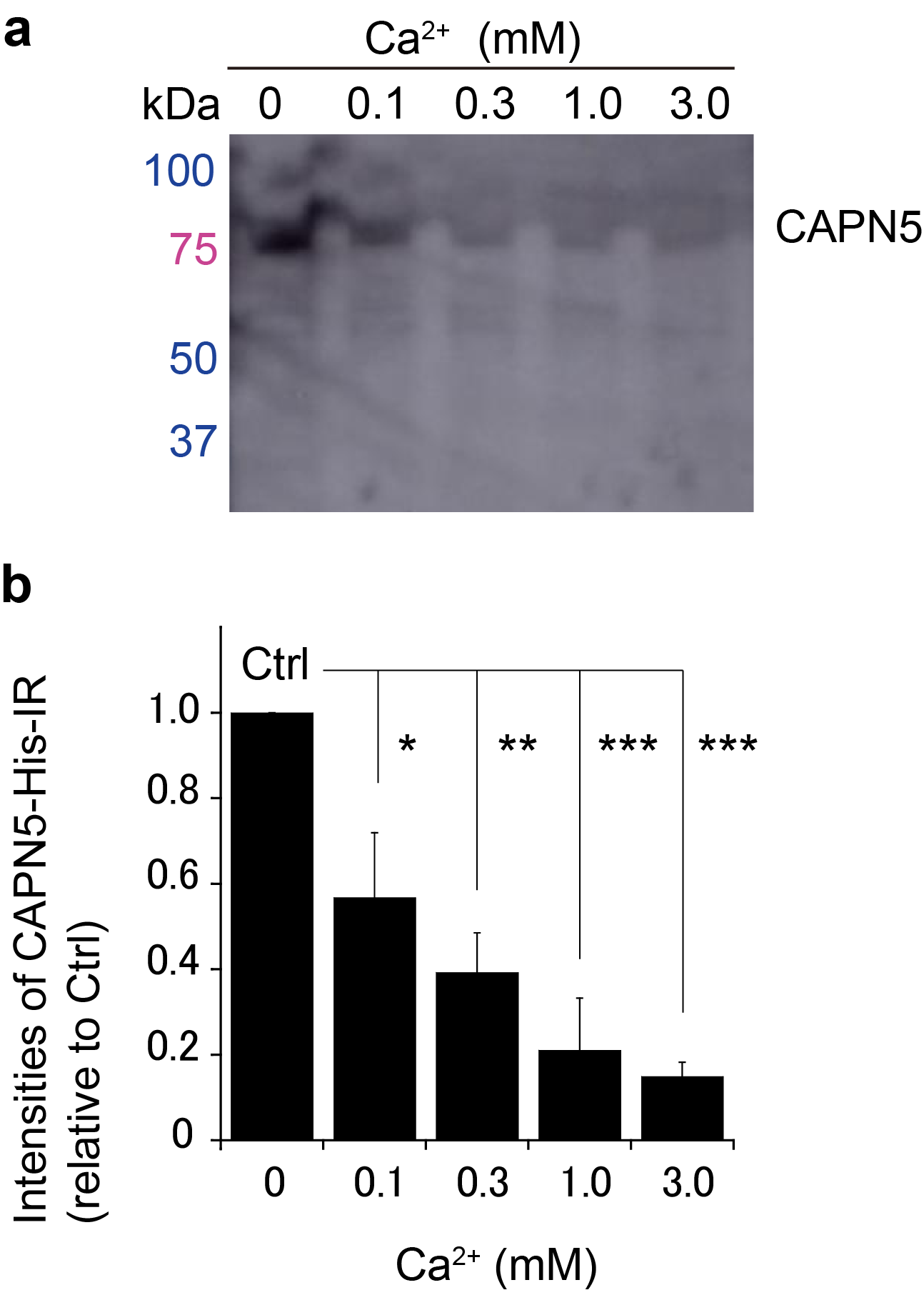
**

**Legend for Supplementary Figure 3**

**The enzymatic activity of CAPN5 was examined by autolytic efficacy.**

**a,** The autolytic activity of CAPN5-His was confirmed by western blot after *in vitro* incubation in the presence or absence of Ca^2+^ (0-3 mM) for 24 h. **b,** Summary of the autolytic efficacy of CAPN5-His (n=3). Mean±SD.*, P<0.01: **, P<0.001, ***P<0.0001.

**STATISTICS**: (**b**) One-way ANOVA with a *post hoc* Tukey‒Kramer comparison among different Ca^2+^-concentration groups (0-3 mM): F(4,10)=36.712, P<0.0001: Tukey‒Kramer test; P_(Ctrl vs 0.1 mM)_=0.0021; P_(Ctrl vs 0.3 mM)_=0.0001; P_(Ctrl vs 1.0 mM)_<0.0001; P_(Ctrl vs 3.0 mM)_<0.0001. The other values from the *post hoc* test are shown in the Supplementary Data. **Supplementary Figure 4**

**
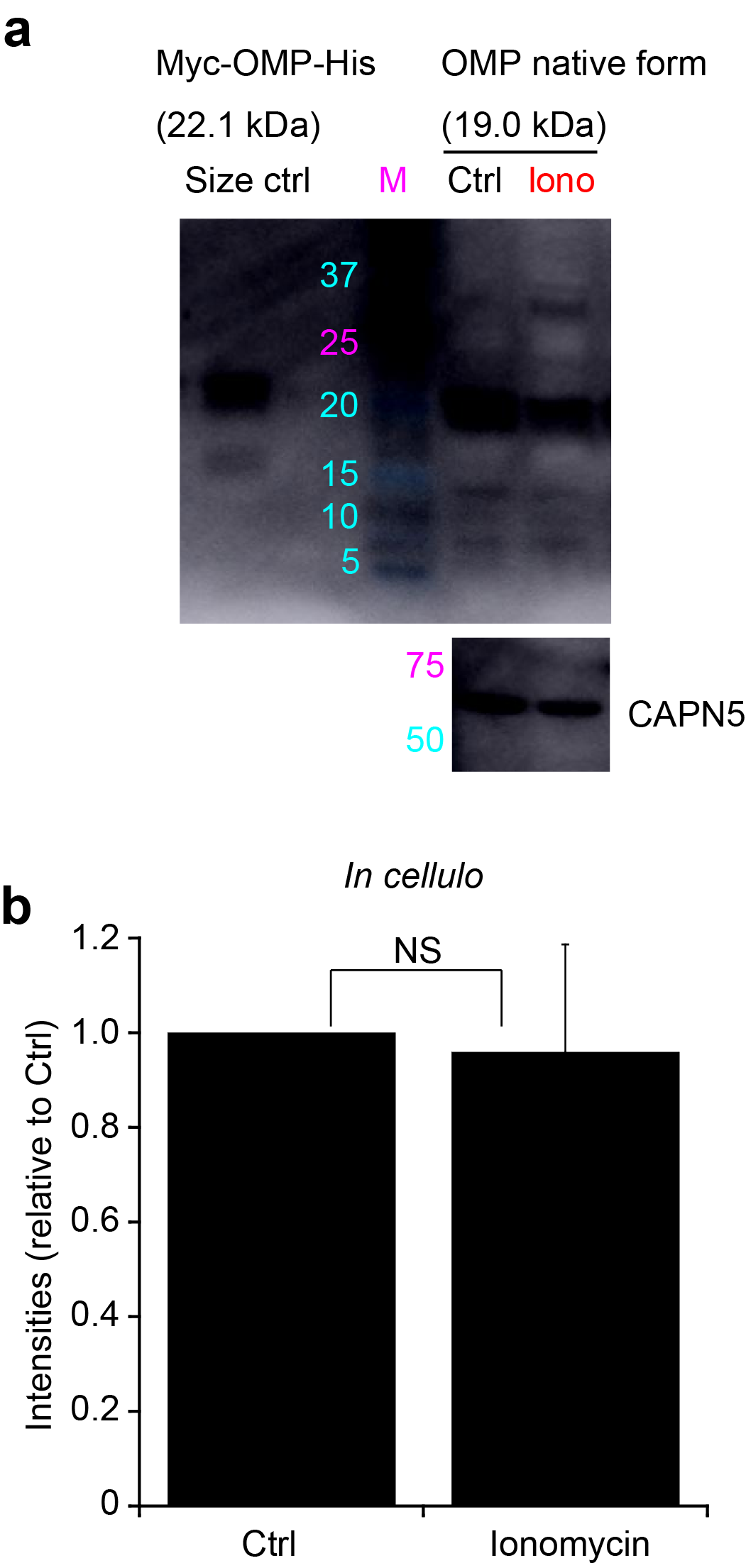
**

**Legend for Supplementary Figure 4**

**The native form of OMP was not cleaved by CAPN5 in HEK293T cells.**

**a,** Ionomycin treatment did not apparently change the OMP-IR of the native-form OMP (19 kDa) expressed in HEK293T cells together with CAPN5-His, whose enzymatic activity was confirmed *in vitro* (Supplementary Fig. 3). **b,** Summary of the native-form OMP-IR (n=3). No significant difference was detected (NS). Mean±SD.

**STATISTICS**: (**b**) Paired, two-tailed Student’s T test for the comparison between Ctrl and ionomycin; P = 0.953.

**Supplementary Figure 5**

**
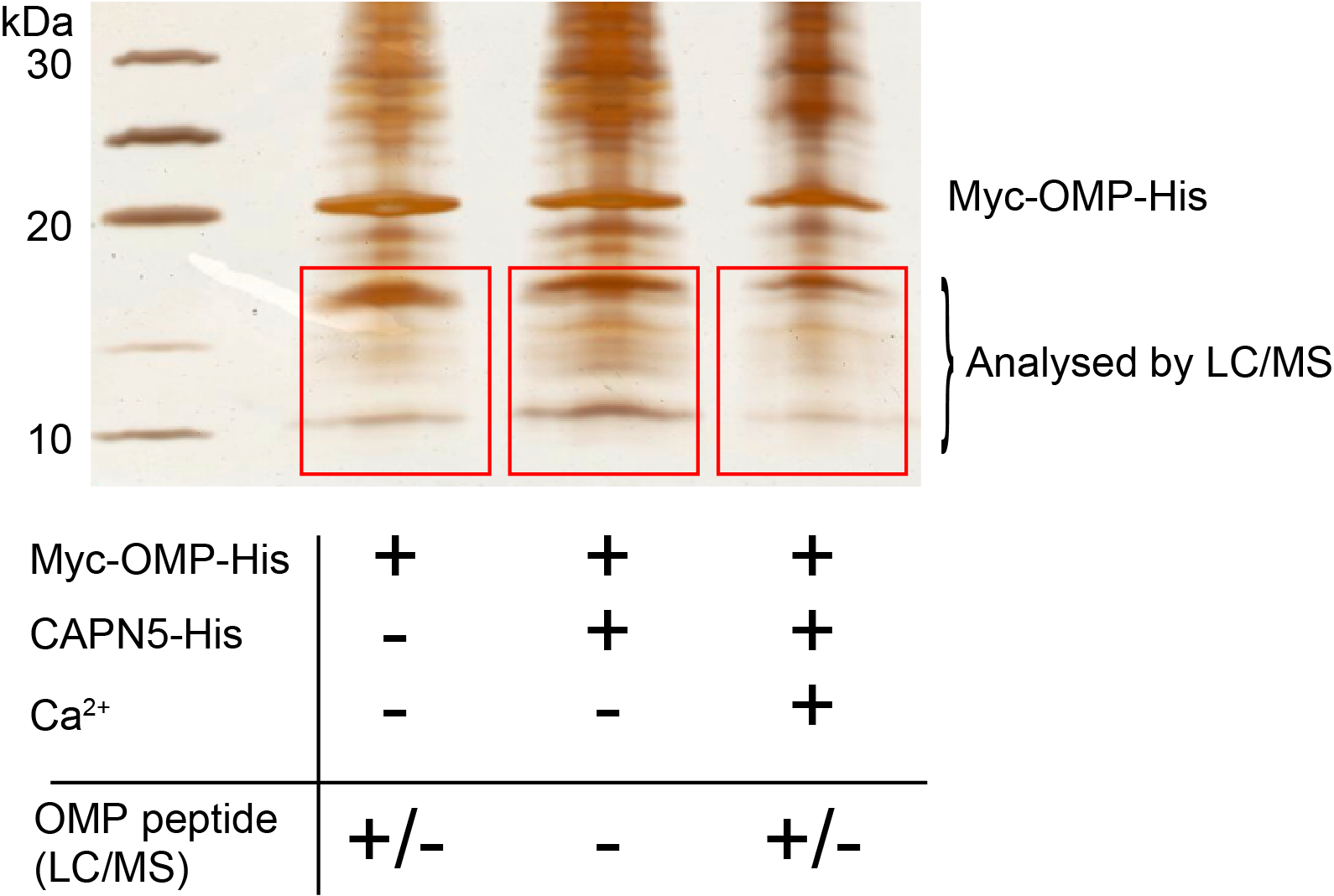
**

**Legend for Supplementary Figure 5**

**Liquid chromatography‒mass spectrometry (LC/MS) did not detect specific fragmentation of Myc-OMP-His.** Protein smear bands below 17 kDa after electrophoresis were trypsinized. The obtained peptides were analysed by LC/MS and identified with Mascot software. Although the same peptide fragment (LIRPAESVYR) corresponding to OMP was detected in the samples for Myc-OMP-His alone (left lane, control) and for the presence of Myc-OMP-His, CAPN5-His and Ca^2+^ (right lane), the identity scores were both below the theoretical criteria to be considered significant (+/-) in the MASCOT search. No other OMP-derived peptides trypsinized with arginine or lysine in the C-termini were detected in any of the samples. Thus, we concluded that this LIRPAESVYR fragment was derived from the contamination of smaller fragments of Myc-OMP-His within the His-tagged purified solution. The other detected peptides mostly corresponded to protein synthesis, indicating that the OMP-associated proteins under translation were obtained by His-tag purification. See also Supplementary Data.

**Supplementary Figure 6**

**
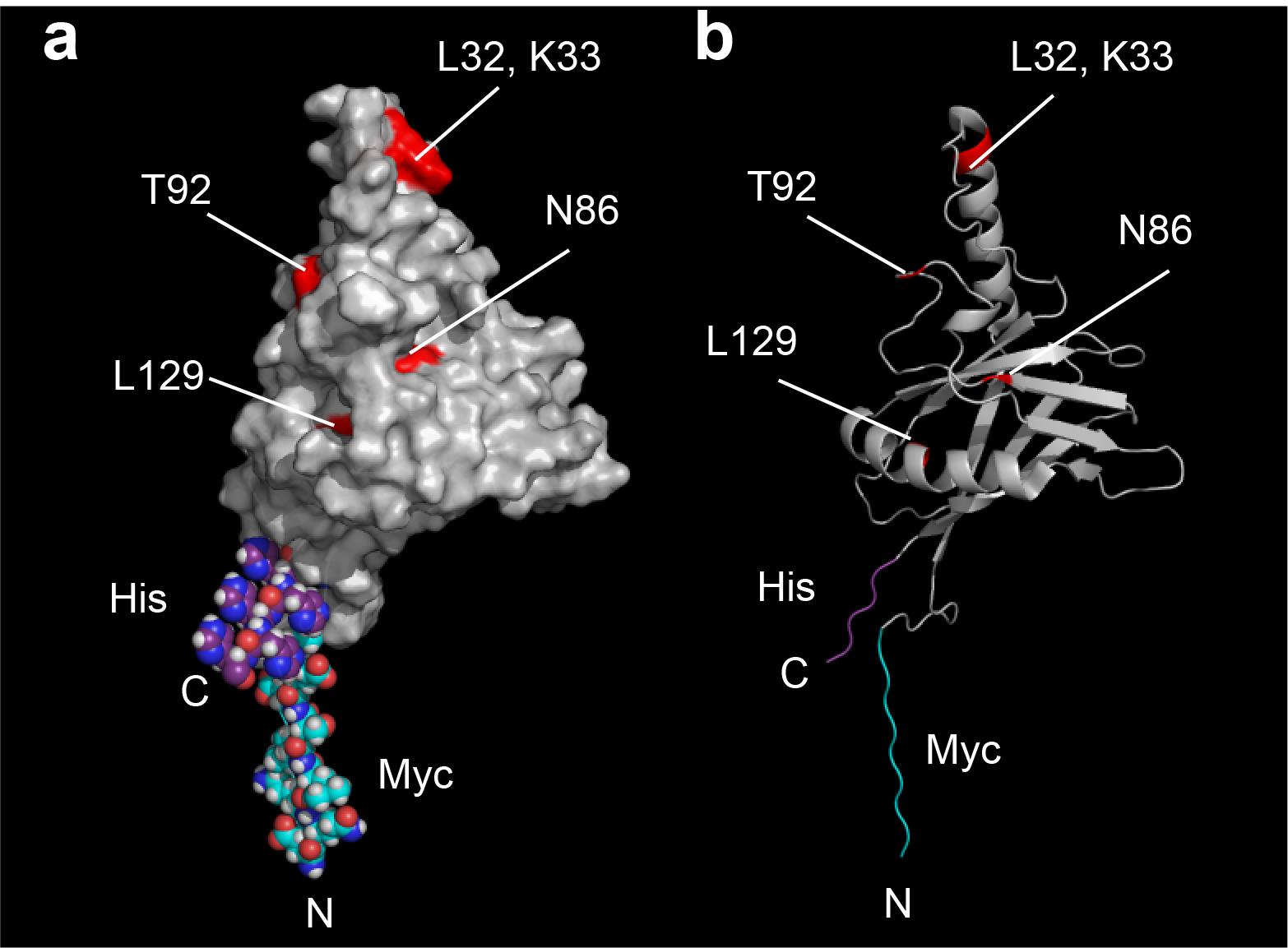
**

**Legend for Supplementary Figure 6**

**Myc- and His-tags were located apart from the expected cleavage sites.**

**a, b,** Crystal structure of OMP fused with Myc- and His-tags to the N- and C-termini in (a) surface model and (b) ribbon-model. The calpain cleavage candidates expected by two or more models in Figure 1a are coloured red.

**Materials and Methods**

**Animals**

We treated experimental animals in accordance with the Kurume University guidelines. C57BL/6 mice were purchased from SLC (Japan SLC, Inc., Shizuoka, Japan). The 8-week-old male mice used in experiments were anaesthetized by intraperitoneal injection of dexmedetomidine, midazolam and butorphanol (4, 10 and 0.5, in mg/kg, respectively) before rapid decapitation with sharp blades for extraction of mRNA as described below or before fixation by perfusion with 4% paraformaldehyde solution (FUJIFILM Wako Pure Chemical Corporation, Osaka, Japan) for immunohistochemistry as described below.

**Cloning of OMP and CAPN5**

Anaesthetized mice were decapitated, and the brain was dissolved in the appropriate solution to extract mRNAs using a kit (Roche, Basel, Switzerland). A cDNA mixture was synthesized from the mRNA using Superscript IV (Invitrogen, CA, USA). The cDNAs for OMP (NM_011010.2) and CAPN5 (NM_007602) were subjected to 35 cycles of 98°C for 10 s, 64°C for 20 s, and 72°C for 120 s using Platinum SuperFi II DNA Polymerase (Invitrogen, CA, USA), and the amplified cDNAs were cloned into the pCI mammalian expression vector by using the In-Fusion HD Cloning Kit (Takara Bio Inc.). Myc-tag immediately after the start codon (peptide, EQKLISEEDL: DNA sequence GAA CAA AAA TTG ATA TCG GAG GAA GAT TTA) and His-tag immediately before the stop codon (peptide, HHHHHH: DNA, CAC CAC CAC CAC CAT CAT CAT CAT) were synthesized with primers. To confirm CAPN5-IR and His-IR expression by using mus anti-CAPN5 and anti-His antibodies, CAPN5-His was subcloned into pIRES2-EGFP (Clontech, CA, USA) to coexpress GFP. CAPN5-IR and His-IR both colocalized with GFP fluorescence, which indicated that CAPN5 was successfully tagged with His.

**Heterologous cDNA expression**

HEK293T cells (ATCC, VA, USA) were cultured in Dulbecco’s modified Eagle’s medium (DMEM; Wako Pure Chemical, Osaka, Japan) supplemented with 10% foetal bovine serum (FBS: Sigma‒Aldrich, MO, USA) without antibiotics at 37°C in 5% CO_2_. HEK293T cells were plated on a cover slip (Matsunami Glass Ind., Ltd., Osaka, Japan) and transfected with 1 µg per well of total plasmids using polyethylenimine (Linear, MW 25000, Transfection Grade, Polysciences, Inc., PA, USA)[1]. The transfected cells were incubated for 24 h for subsequent immunocytochemistry or protein extraction.

**Fluorescence immunohistochemistry and immunocytochemistry**

For immunocytochemistry, the transfected HEK293T cells on cover slips were immersion-fixed in ice-cold 4% formaldehyde in divalent free PBS for 5 minutes at 25°C, washed with PBS three times for more than 30 minutes total, incubated with the primary antibodies in blocking solution (Nacalai Tesque, Kyoto, Japan) at 25°C for 6 hours, washed with PBS three times and incubated with the appropriate secondary antibodies (1:200; Alexa Fluor 488-conjugated anti-rabbit, ab150061; Alexa Fluor 594-conjugated anti-rabbit, ab150064; Alexa Fluor 488-conjugated anti-mouse, ab150109; Alexa Fluor 594-conjugated anti-mouse, ab150108; Alexa Fluor 594-conjugated anti-goat, ab150136; all from Abcam, Abcam, Cambridge, UK) with 4',6-diamidino-2-phenylindole (diluted 1:1000; Nacalai Tesque) in PBS with 0.5% Triton-X at 24°C for 1 hour. Primary antibodies against Myc (rabbit; Ab9106, Abcam, Cambridge, UK) [2], His (mouse 66005-1-Ig, Proteintech, IL, USA)[3], OMP (goat; 019-22291, FUJIFILM Wako Pure Chemical Corporation, Osaka, Japan)[4], and CAPN5 (mouse; sc-271271, Santa Cruz Biotechnology, TX, USA)[5] were used in the present study. The cover slips were then rinsed in distilled water, dried, mounted on MAS-coated glass slides (Matsunami Glass Ind., Ltd., Osaka, Japan) using Vectashield antifade reagent (Vector Labs, CA, USA) and tightly sealed. For immunohistochemistry, tissues after perfusion fixation were postfixed for 10 h, cryoprotected by overnight incubation in PBS containing 30% w/v sucrose at 4°C, mounted in OCT Embedding Compound (Sakura Finetek, Tokyo, Japan) and frontally sectioned at a 30-μm thickness using a cryostat (CM3050S, Leica Microsystems, Wetzlar, Germany) at -20°C. The sections were then incubated at 25°C overnight in a blocking solution containing the following primary antibodies: OMP (goat; 019-22291, FUJIFILM Wako Pure Chemical Corporation) and CAPN5 (mouse; sc-271271, Santa Cruz Biotechnology). The samples were washed with PBS and incubated in PBS with Triton X-100 (FUJIFILM Wako Pure Chemical Corporation), 4',6-diamidino-2-phenylindole (diluted 1:1000; Nacalai Tesque) and the anti-IgG secondary antibodies (diluted 1:200; Alexa Fluor 594-conjugated anti-goat and Alexa Fluor 488-conjugated anti-mouse) for 1.5-2 h. The samples were then washed in PBS, mounted onto MAS-coated glass slides (Matsunami Glass, Tokyo, Japan), coverslipped using Vectashield antifade reagent (Vector Labs, CA, USA) and tightly sealed. Heat-mediated antigen retrieval was performed prior to reaction with the CAPN5 antibody by incubating the fixed sample at 95°C in 1 M Tris-HCl (pH 8.0; Nacalai Tesque) for 15 min. Fluorescence signals were detected using a fluorescence microscope (BX50, Olympus, Tokyo Japan), imaged with a digital camera (DP72, Olympus) and analysed with cellSens image analysis software (Olympus).

**Reaction of OMP and CAPN5 *in vitro* and *in cellulo***

cDNAs for Myc-OMP-His and CAPN5-His were heterologously expressed in HEK293T cells. The His-tagged proteins were purified by using a Capturem His-Tagged Purification Kit (Takara Bio USA, Inc.). The solution was replaced with PBS without divalent cations (PBS(-)) by using centrifugal ultrafiltration units (Amicon Ultra15 10,000 NMWL, Merck Millipore, MA, USA). The protein concentration was confirmed with a NanoDrop Lite (Thermo Fisher Scientific, MA, USA). We mixed OMP and CAPN5 at concentrations of 1 μg/μL and 0.1 μg/μL, respectively, in 100 μL of reaction mixture and different concentrations of CaCl_2_ (0-3 mM), which were incubated at 37°C for 3 h or 24 h. To load Ca^2+^ *in cellulo*, we transfected HEK293T cells with Myc-OMP-His and CAPN5-His as described above and incubated them in the absence or presence of ionomycin (1 μM; Cayman Chemical, MI, USA) for 12 hours. After the reactions, the cells were collected, resuspended in PBS(-) and lysed by sonication at 0°C. The whole-cell lysate was subjected to western blotting.

Western blotting

After the reactions, the solutions were mixed with Laemmli sample buffer (Bio-Rad Laboratories, CA, USA) at 25°C, supplemented with 10% 2-mercaptoethanol (Sigma‒Aldrich, MO, USA), heated at 95°C for 5 min and used for subsequent western blotting. Then, 5-μL volumes of the samples were electrophoresed using a precast, 4-20% Mini-PROTEAN TGX gel (Bio-Rad Laboratories) in a Mini-PROTEAN Tetra cell (Bio-Rad Laboratories) according to the manufacturer’s instructions. Then, the proteins were transferred to PVDF membranes (Midi Format 0.2 µm PVDF Cat. #1704157, Bio-Rad Laboratories, CA, USA) using a Trans-Blot Turbo Transfer System (Bio-Rad Laboratories, CA, USA) for 7 min at 25 V. The PVDF membranes were incubated with primary antibodies against Myc (rabbit; Ab9106, Abcam), OMP (goat; 019-22291, FUJIFILM Wako Pure Chemical Corporation), His (mouse; 666005-1-Ig, Proteintech) or CAPN5 (mouse; sc-271271, Santa Cruz Biotechnology, (all 1:1000) diluted with Can Get Signal Immunoreaction Enhancer Solution 1 (NKB-101, Toyobo, Osaka, Japan) overnight at 4°C and washed with 0.1% Tween in Tris-buffered saline (TBST: 10 mM Tris; 150 mM NaCl; pH 7.6) 3 times for 10 minutes each. The membranes were incubated with an anti-rabbit secondary antibody conjugated with horseradish peroxidase (HRP; 7074P2, Cell Signaling Technology, MA, USA), an anti-goat HRP-conjugated secondary antibody (ab6885, Abcam) or an anti-mouse HRP-conjugated secondary antibody (ab6823, Abcam, Cambridge, UK) all at a 1:2000 dilution in Can Get Signal Immunoreaction Enhancer Solution 2 (NKB-301, Toyobo) for 1.5 h at 25°C and then washed with TBST 3 times for 10 minutes each. Then, the membranes were washed carefully. Immunoreactivity was detected using ECL Prime western blot detection reagent (RPN2232, Cytiva, NJ, USA). The images were captured using an Amersham Imager 600 (GE Healthcare Biosciences, NJ, USA). To detect several immunoreactivities, the membranes were stripped of the first set of primary and secondary antibodies by using WB Stripping Solution (05364-55, Nacalai Tesque) and sequentially reprobed with the next set of primary and secondary antibodies.

**Nanoscale liquid chromatography-tandem mass spectrometry (nanoLC/MS)**

NanoLC/MS analysis was performed by Japan Proteomics Co. Ltd. (Miyagi, Japan) by using the facility’s equipment and reagents. Briefly, the gel after SDS‒PAGE was stained using a silver stain MS kit (FUJIFILM Wako Pure Chemical Corporation). The gel slippage was reduced by 100 mM dithiothreitol and alkylated by 100 mM iodoacetamide. After washing, the gels were incubated with trypsin overnight at 30°C. Recovered peptides were desalted by ZipTip C_18_ (Merck Millipore). Samples were analysed by nanoLC/MS systems (DiNa HPLC system, KYA TECH Corporation, Tokyo, Japan; QSTAR XL, Applied Biosystems, MA, USA). Mass spectroscopy data acquisitions were piloted by Mascot software (Matrix Science, London, UK) to match proteins in the database.

**Structural model simulation**

We used PyMOL (The PyMOL Molecular Graphics System, Version 2.0 Schrödinger, LLC., NY, USA) and AutoDock Vina[21] (The Scripps Research Institute, CA, USA) for structural modelling. We obtained the crystal structures of OMP[20] (1zri) from the Protein Data Bank (PDB). The protein data were further modified by adding polar hydrogen atoms and rendered into an analysis grid using AutoDock Tools (version 1.5.6)[21]. Selenomethionine (MSE in the PDB data) was not edited. Myc- and His-tags (EQKLISEEDL and HHHHHH, respectively, in the single-letter amino acid code) were fused to the N- and C-termini of the OMP structure model in the PyMOL platform.

**Prediction**

We consulted three prediction online software programs: Calpacchopper[6][7] used three models and suggested several different candidate residues, while DeepCalpain[8] and SitePrediction[9] suggested no calpain cleavage sites for all the available calpains in mouse OMP.

**Statistical analyses**

Statistical analyses were performed using either Microsoft Excel (Microsoft, WA, USA) or KaleidaGraph 4 (Synergy Software, PA, USA). Detailed statistical methods, values, and numbers of experimental samples are provided in the Supplemental Datasheet.

**Supplementary References**

1. Durocher Y, Perret S, Kamen A. High-level and high-throughput recombinant protein production by transient transfection of suspension-growing human 293-EBNA1 cells. Nucleic Acids Res. 2002;30:1–9.

2. Hatzopoulos GN, Kükenshöner T, Banterle N, Favez T, Flückiger I, Hamel V, et al. Tuning SAS-6 architecture with monobodies impairs distinct steps of centriole assembly. Nat Commun. Springer US; 2021;12:3805.

3. Wang Z, Sheng C, Yao C, Chen H, Wang D, Chen S. The EF-Hand Protein CALML6 Suppresses Antiviral Innate Immunity by Impairing IRF3 Dimerization. Cell Rep. 2019;26:1273-1285.e5.

4. Nakashima N, Nakashima K, Taura A, Takaku-Nakashima A, Ohmori H, Takano M. Olfactory marker protein directly buffers cAMP to avoid depolarization-induced silencing of olfactory receptor neurons. Nat Commun. 2020;11:2188.

5. Schaefer KA, Toral MA, Velez G, Cox AJ, Baker SA, Borcherding NC, et al. Calpain-5 expression in the retina localizes to photoreceptor synapses. Investig Ophthalmol Vis Sci. 2016;57:2509–21.

6. Shinkai-Ouchi F, Koyama S, Ono Y, Hata S, Ojima K, Shindo M, et al. Predictions of cleavability of calpain proteolysis by quantitative structure-activity relationship analysis using newly determined cleavage sites and catalytic efficiencies of an oligopeptide array. Mol Cell Proteomics. 2016;15:1262–80.

7. duVerle DA, Ono Y, Sorimachi H, Mamitsuka H. Calpain Cleavage Prediction Using Multiple Kernel Learning. Schönbach C, editor. PLoS One. 2011;6:e19035.

8. Liu ZX, Yu K, Dong J, Zhao L, Liu Z, Zhang Q, et al. Precise prediction of calpain cleavage sites and their aberrance caused by mutations in cancer. Front Genet. 2019;10:1–13.

9. Verspurten J, Gevaert K, Declercq W, Vandenabeele P. SitePredicting the cleavage of proteinase substrates. Trends Biochem Sci. 2009;34:319–23.
